# Differences and similarities in muscle architecture of fibularis longus and brevis—An observational descriptive cross-sectional and feasibility study

**DOI:** 10.1186/s13018-024-04594-2

**Published:** 2024-02-01

**Authors:** Anna E. Sprinchorn, Norman Eizenberg, Priscilla J. Barker

**Affiliations:** Uppsala University, Uppsala, Sweden

**Keywords:** Fibularis longus, Fibularis brevis, Muscle architecture lower limb

## Abstract

**Background:**

The fibularis longus (FL) muscle is larger in volume than fibularis brevis (FB) and is therefore claimed to be the stronger evertor of the two. Clinical observation of FL and FB tendon rupture show that injury to the FB has a serious negative effect on hindfoot eversion. This implies that the FB is the stronger and more important evertor. The strength of a muscle is not purely based on its volume, and the observed discrepancy between the FB and FL may be due to differences in muscle architecture. This study compares the muscle architecture of FL with FB.

**Methods:**

Sixteen legs from eight formaldehyde-fixed human specimens, mean age 83 (range 72–89) years, were dissected. The volume, fibre lengths and fibre pennation angles for both muscles were measured and the physiological cross-sectional area (PCSA) was calculated.

**Results:**

The FL was always larger than the FB, with an individual difference in volume that varied from 1.4 to 4.6 times larger with a mean difference of 17 ml (95% CI 14–20; *p* < 0.001). Mean fibre lengths were 9 mm (95% CI 2–16; *p* = 0.015) longer in FL than in FB. The mean pennation angle was 9.6 degrees in FL and 8.8 degrees in FB, this difference was not significant (*p* = 0.32). The mean PCSA for FL was 3 cm^2^ (95% CI 2–4) larger than for FB (*p* < 0.001).

**Conclusions:**

With our sample set, the hypothesis that the muscle architecture can explain the clinical discrepancy between the FL and FB, was not supported. The difference in hindfoot eversion might instead depend on the different moment arms of FL and FB and the effect forefoot abduction has on hindfoot eversion.

## Introduction

Both fibularis (previously known as peroneus) muscles, described as evertors of the foot [1], risk tendon rupture during supination injury of the ankle [2] as well as in the case of ligamental laxity [3]. Traditionally the fibularis longus (FL) is claimed to be a stronger evertor than fibularis brevis (FB) [4–6], based on anatomical studies describing the FL as the larger muscle of the two [4, 7]. However, in clinical practice, a tendon rupture of the FB more often leads to the patients developing a symptomatic hindfoot varus than a FL tendon rupture [8, 9] due to the weakness in muscle strength. There are also reports of surgical procedures where harvesting of the FL tendon has shown no adverse effects on foot function [10–12]. The discrepancy in size and work output in the FL might be related to the muscle architecture. The mass and volume of a muscle can be a poor predictor of its function and muscle strength [13] since the work output of a muscle is not only related to the size but also affected by the arrangement of the muscle fibres (muscle architecture) [14–17]. This anatomical study determines the difference in size between FL and FB and explores the similarities and differences in muscle architecture between the two. The focus is on the fibre length, pennate angles and the physiological cross-sectional area (PCSA). This study hypothesised that the FL and FB muscles have different muscle architectures, with the FL having longer fibres and more significant displacement potential, while the FB muscle has shorter fibres and higher pennation angles, but larger PCSA, thereby having the potential to exert more power [14, 15, 18].

## Methods

**Fig. 1:**
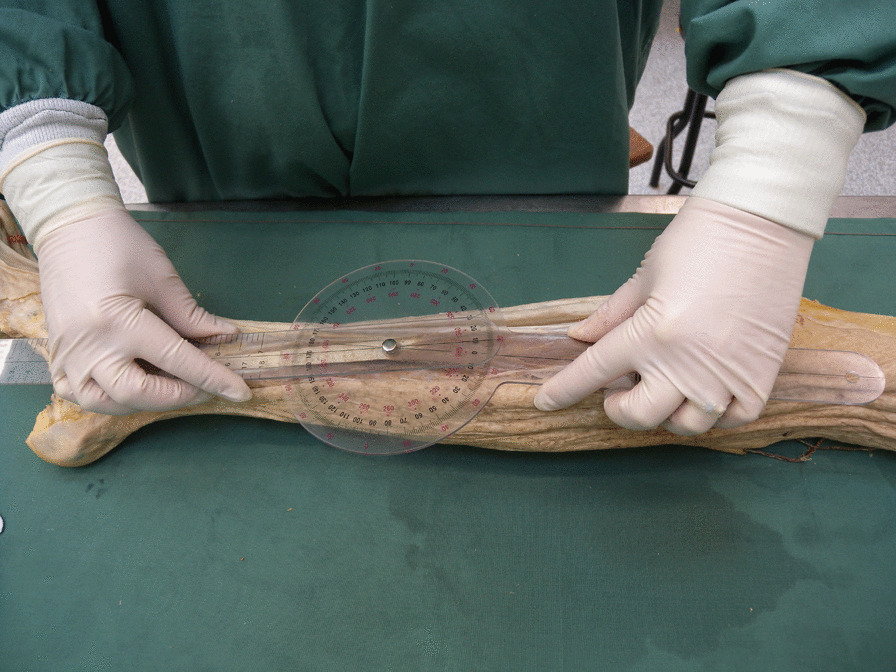
The insertional angle of muscle fibres to the tendon is measured using a goniometer

**Fig. 2:**
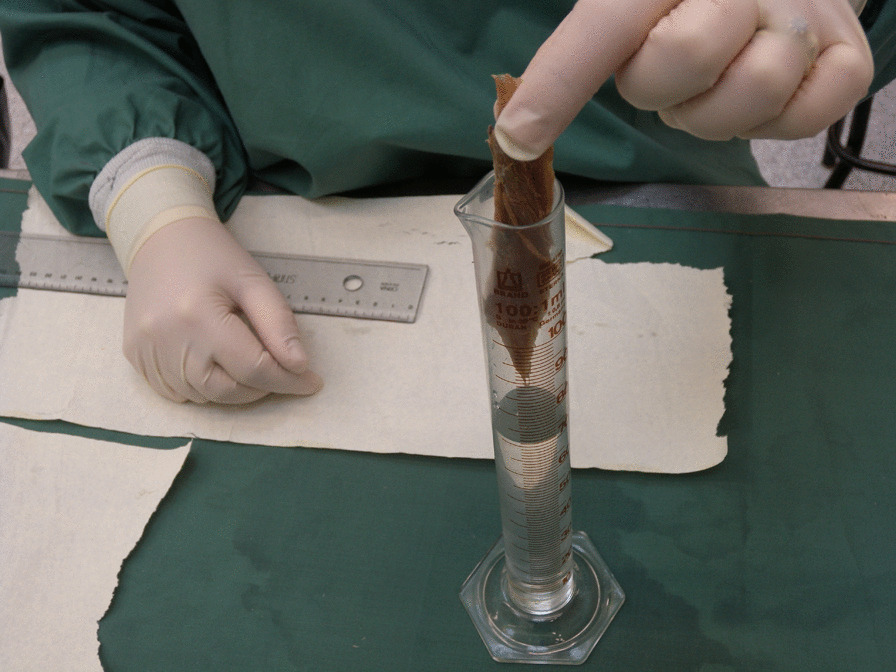
The volume of the muscle fascicles is measured using water displacement

In this observational descriptive cross-sectional and feasibility study, sixteen lower limbs (eight formaldehyde-fixed human specimens) provided by the Department of Anatomy and Developmental Biology, School of Biomedical Sciences, Faculty of Medicine, Monash University, Melbourne, Australia, were dissected. Ethical approval for research on cadaveric material at the Department of Anatomy and Cell Biology is licensed under the Human Tissue Act and was approved by the Monash University. The specimens were all Caucasian, with a median age of 83 years (range 72–89 years) and there were four women and four men. The first author performed the dissections with the assistance of a physiotherapist. The FL was exposed, the fibre angles were measured in situ with a goniometer (Fig. 1), and the attachments were documented. The angle of the deeper muscle fibres was measured after the superficial layers were removed. The FL's muscle fascicles (muscle fibres in bundles) were then reflected from their origin and shred-removed, after which the length and volume of each fascicle were measured. The fascicle length was measured directly with a millimetre scale ruler (accuracy ± 0.1 cm) and the volume of the fascicle was measured by water displacement (± 1 ml accuracy) [19] (Fig. 2). After the FL was removed, the FB was exposed, the attachments documented, and the pennation angle and muscle fascicles were measured the same way as for the FL. The plantar flexion angle of the foot was noted by measuring the angle between the fibula and the fifth metatarsal using a goniometer and the length of the fibula was recorded.

### Volume

The volume for each muscle was calculated by adding the volume of all fascicles for each muscle. All the fascicles in each muscle were then added together and measured with water displacement once more as an extra control of the volume.

### Fibre length

The length of the fascicles was measured to calculate excursion (distance), since this will affect the work output (work = tension × distance).

### Pennation angles

**Fig. 3:**
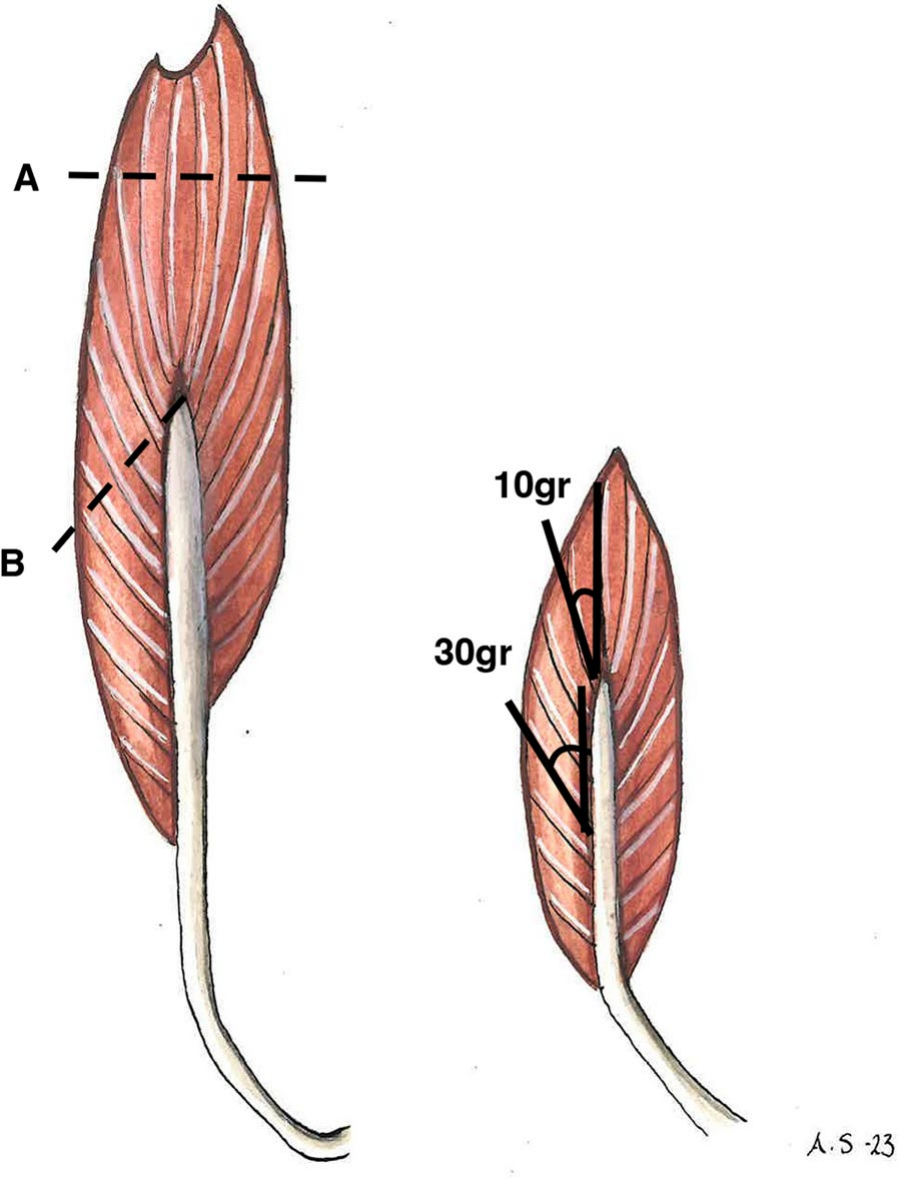
Schematic illustration of the fibularis longus and brevis, illustrating the different physiological cross-sectional areas (PCSA:s) for a fusiform shape (**A**) and pennate muscle (**B**), as well as how the insertional angle for the muscle fibres varies from 10 degrees proximal to 30 degrees distal over the length of the FB tendon

In a pennate muscle the muscle fibres lie at an angle to the line of action. The pennation angles of the fibularis muscles change over the length of the muscle, from mid- to distal portion (Fig. 3). The different angles for the separate muscle fascicles were measured, since both the force of the muscle and the excursion, and in consequence the work output, are affected by the angle of fibres towards the tendon. The force working on the tendon is the force of the muscle times the cosinus of the pennation angle [15]. The higher the angulation, the less distance the muscle can pull the tendon; consequently, the less work output. Parallel-fibred muscles have the most significant number of sarcomeres arranged in series. They can make the most effective shortening of the total distance between the origin and insertion of the muscle tendon unit. In contrast, pennate muscles have greater holding power but less excursion. Pennation allows more and shorter fibres to be packed into the available space [15]. The foot’s position in plantar flexion was recorded since this might affect the pennation angle.

### PCSA

The length of the fascicles was also measured to calculate the PCSA, based on the study by Haxton [14]. The strength of a muscle is not directly related to the muscle volume but rather to the PCSA of the muscle, that is, a section that passes through practically all of the muscle fibres [14, 16, 18, 20]. In a muscle with parallel fibres, the PCSA is the same as the cross-section of the whole muscle. Since both the FL and FB are bipennate muscles, the cross-section has to be calculated by adding the results from the different muscle fascicles (Fig. 3). Approximating the shape of a muscle fascicle to that of a cylinder, the formula volume = cross-sectional area × length could be used [14]. The PCSA was subsequently calculated by dividing the volume by the fibre length and summing the results for all fascicle bundles of the muscle [14, 19].

### Statistical analysis

The differences in total muscle volumes, mean fibre lengths, mean pennation angles and total PCSA between the FL and FB muscles were calculated using fixed effects linear regression. This method was chosen to consider the dependence that is introduced when both sides (right and left legs) were used for all measurements. Spearman’s coefficient was used to correlate the length of the fibula to the volume of the fibularis longus.

## Results

### Volume

The FL was larger than the FB in all legs, and the difference varied from 1.4 to 4.6 times larger. The mean difference in size between FL and FB (in the same specimen) was 2.4 (SD 1.0); the median was 2.2 (range, 1.4–4.6). The mean volume of FL was 32 ml (SD 13) and the mean volume of FB was 15 ml (SD 9) (Table 1) with a mean difference of 17 ml (95% CI 14–20; *p* < 0.001). The volume of FL had a weak positive correlation to the length of the fibula (Spearman’s coefficient r_s_ = 0.35).

**Table 1:** Summary of results of the total volume, average fibre lengths, average pennation angles and total PCSA

	Volume ml Mean	Volume ml Median	Fibre length mm Mean	Fibre length mm Median	Pennation angle degrees Mean	Pennation angle degrees Median	PSCA cm^2^ Mean	PCSA cm^2^ Median
FL	32 (SD 13)	33 (range, 11–58)	58 (SD 19.3)	51 (range, 30–130)	9.6 (SD 7.6)	10 (range, 0–30)	5.8 (SD 2.9)	5.0 (range, 1.5–11.7)
FB	15 (SD 9)	15 (range, 5–30)	49 (SD 19.5)	42 (range, 20–111)	8.8 (SD 6.3)	8 (range, 0–25)	3.1 (SD 1.4)	3.4 (range, 0.7–5.4)

### Fibre length

The mean average fibre length was 58 mm (SD 19.3) for FL and for FB it was 49 mm (SD 19.5) (Table 1). The FL had longer fibres than FB except for one specimen (two legs). Mean fibre lengths were, on average, 9 mm (95% CI 2–16; *p* = 0.015) longer in FL than FB.

### Pennation angle

The mean average pennation angle was 9.6 degrees (SD 7.6) for FL and 8.8 degrees (SD 6.3) for FB (Table 1) and this difference was not significant (*p* = 0.32). The mean difference between the angles of the FL and FB was 0.8 degrees with a 95% confidence interval of (− 0.8, 2.4). Within each muscle the angle varied from 0 degrees at the proximal part to up to 30 degrees at the distal portion of the muscle for the FL and 25 degrees for the FB*.* All the specimens had their feet in plantar flexion, ranging from 15 to 45 degrees, with a mean value of 35 (SD 9.2) degrees. The median in plantar flexion was 37 (range, 15–45) degrees since one specimen was an outlier with less plantar flexion than the rest.

### PSCA

The mean PCSA for FL was 5.8 cm^2^ (SD 2.9) and for FB 3.1 cm^2^ (SD 1.4) (Table 1). The mean PCSA for FL was 3 cm^2^ (95% CI 2–4) larger than for FB (*p* < 0.001). The mean difference in PCSA between the FL and FB (in the same specimen) was 1.9 (SD 0.7).

## Discussion

**Fig. 4:**
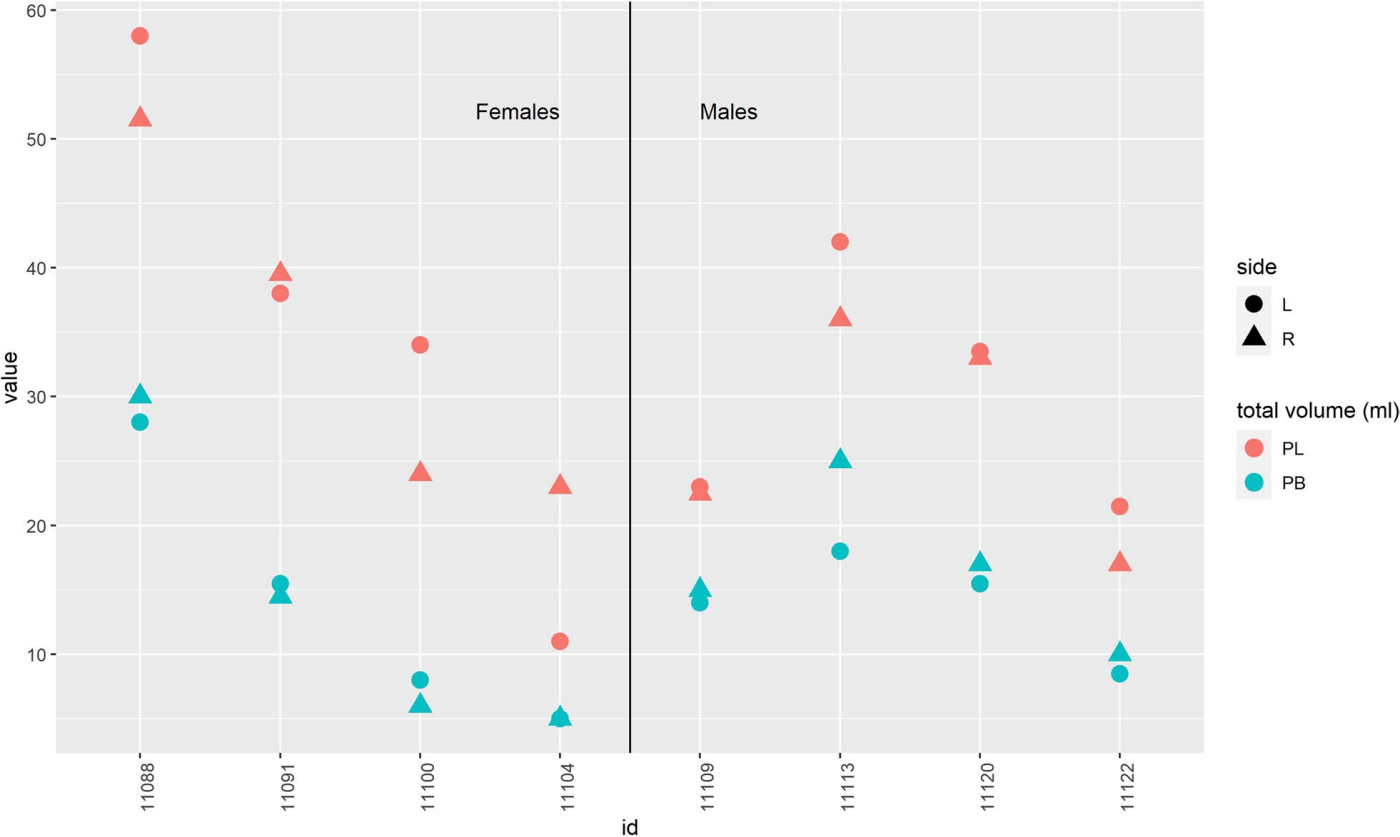
The difference in volume (ml) between fibularis longus (FL, red) and fibularis brevis (FB, blue)

**Fig. 5:**
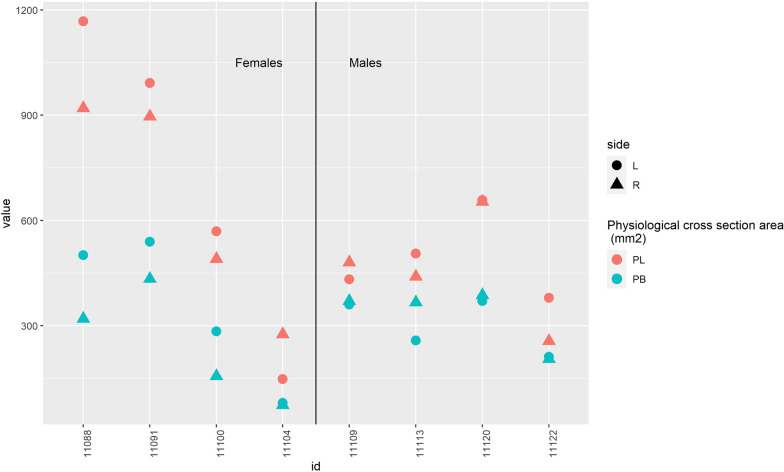
The difference in PCSA (mm^2^) between fibularis longus (FL, red) and fibularis brevis (FB, blue)

This observational descriptive cross-sectional and feasibility study concludes, as has been stated in previous studies, that the FL has a larger volume and longer fibre length than FB, theoretically leading to higher excursion and higher work output for FL [18]. The study further establishes that the two muscles have very moderate difference in pennation angle. Both the FL and FB have a proximal part with a fusiform shape, which affects the mean pennation angle. The two muscles have no significant difference in their muscle architecture and subsequently the holding power (tension) is not more significant for FB compared with FL. Although this non-significant difference might have been significant with a larger sample, the magnitude of the difference in pennation angles is too small to affect the work output. The hypothesis was that the FB due to a significant difference in muscle architecture would prove to be the stronger despite its lower volume, but this could not be proven. The volume of a muscle will affect its power—but the power of a muscle also depends on its cross-sectional area and not its length [14]. The analysis shows that the difference in PCSA between FL and FB is smaller in comparison to their difference in volume (Figs. 4 and 5). This difference in PCSA is still not enough to explain the clinical importance of FB on the hindfoot stability and the FL remains the stronger muscle with its higher PCSA.

Earlier studies report a PCSA of FL as 10.4 ± 3.8 cm^2^ and for FB 4.9 ± 2.0 cm^2^ [13], based on 21 legs, and another study showed the PCSA for FL as 13 ± 2 cm^2^ and for FB as 7 ± 1 cm^2^, based on eight legs (only men) [19]. The present study reports a smaller PCSA for the FL and FB muscles than has previously been reported. On the other hand, this study reports a longer muscle fibre length than has been found in previous studies. Sopher et al. [20] reported mean fibre length as 37 (± 3) mm for FL and 34 (± 2) mm for FB (eight legs, formalin embalmed). In their study of three legs (formalin embalmed), Wickiewicz et al. [16] noted the FL and FB to have the same muscle fibre length, 39 mm*.* Friederich and Brand [21] reported from two specimens (formalin embalmed) the mean length for FL as 42.6 (SD 0.85) mm and 46.0 (SD 1.77) mm and for FB 35.7 (SD 0.95) mm and 43.4 (SD 1.31) mm, respectively. The relative work capacity of a muscle can be calculated as work = tension × distance. The distance is the excursion of a muscle and is proportional to the length of the fibres [22, 23], and the longer the fibres, the greater the excursion of the muscle [18]. Hintermann et al. have studied the excursion of the fibularis tendons around the ankle joint [23] and report the total excursion as follows: FL: 30.1 mm and FB: 27.5 mm. Haines states that the excursion is 0.57 × fibre length in a stretched state [22]. Using these numbers, the excursion for FL in the present study is calculated as 59 × 0.57 = 33.0 mm for FL and 50 × 0.57 = 28.5 mm for FB, which is very close to the results by Hintermann et al. Some allowance has to be made for the pennation of the muscle fibres, although the angle never exceeded 30 degrees in any specimen in the present study. Even if an increase in the angle will decrease the work output, the effect will only markedly increase once the angle exceeds 30 degrees [15, 18].

Previous reports on pennation angles for the FL and FB show quite some variation: based on three legs, the pennation angle of the FL was 10 degrees contrasting with the five degrees of the FB [16]. Based on 21 legs the average pennation angle for FL was 14.1 degrees, and for FB, 11.5 degrees [13]. No study reports an insertional angle of more than 30 degrees. The angle varies depending on the length of the muscle, and therefore it is challenging to decide on an exact pennation angle for the FL and FB muscles (Fig. 3). Since the two muscles have the same shape and similar mean pennation angle, we conclude that they work in the same way.

The embalmed feet were all in plantar flexion, which might affect the pennation angle. In a pennate muscle, the fibres will rotate around their origin as the muscle contracts, changing the angle of pennation as they shorten [15]. The length varies if the leg is fixated with the muscles in contraction or extension [18]. The full plantar flexion in this age group should be approximately 40 degrees of plantar flexion [24], indicating the specimens had their feet close to full plantar flexion, where the muscles would be at their shortest. If the muscles were at their shortest, the PCSA should be multiplied by the cosine of the angle, giving the “reduced cross-section area” [14], but the effect of the angle is negligible, since the cosine of 9 degrees is 0.98. It is also probable that the feet had passively adopted into the plantar flexed position and the muscles were in rest of tension, so one might assume that muscle fibres were in a relaxed neutral position*.* This study compared FL with FB, so most importantly, both muscles were examined similarly.

There was significant anatomical dispersion in the size of the fibularis muscles, even between the right and left leg within the same specimen. This has been noted by other researchers [13] and accordingly one should be careful to draw any firm conclusions from studies based on a limited number of specimens. A limitation to the present study is the material of only 16 legs, and even if this is a larger material than in most earlier studies, given the anatomical variation shown, a more extensive study would provide more detailed understanding of the differences in FL and FB muscle architecture. Another limitation of the study is the advanced age of the specimens, 72–89 years, which might affect the results in comparison to a younger population. The FL was observed to be the larger of the two muscles in all the specimens and this has been the conclusion of all anatomical studies so far [4, 16, 21]. Only one study, where the calculation of volume is based on hand tracing 20 legs from MRI reports the FB as the larger of the two [25].

Skeletal muscle contractile properties depend not only on muscle volume and muscle architecture but also on muscle fibre physiological properties [17]. The FL and FB are both innervated by the superficial fibular nerve but there are no reports on whether they have the same proportion of slow-contracting fibres (type I) and fast-contracting fibres (type II). In their study of six legs from six specimens, Johnson et al. found 62.5% type I fibres in FL but did not measure the FB [26]. Yang and Yoon reported 40.8% type I fibres in FL, based on 15 specimens [27], but no reports on the FB. The effect of the muscle fibre type is therefore unknown, but fibre properties only slightly influence skeletal muscle function [16, 17] and are primarily based on muscle architectural properties (17).

The present study shows that muscle architecture does not explain why the small FB muscle can hold the eversion in the hindfoot in patients without a working FL, as has been clinically noted [9–12]. The answer to this discrepancy must lay elsewhere, and a reasonable theory is the difference in moment arms. The work of a muscle–tendon unit (MTU) is not only governed by the volume and arrangement of the muscle fibres but also by the moment arm (MA) and the leverage of that MTU about a joint at different joint angles. Work is calculated as force × distance, as mentioned earlier, and this formula is also valid for the lever arms around the foot and ankle, where the moment equals force × the moment arm (*M* = FxMA). While the force is equal along the length of a muscle, its effect might differ as it passes over different joints. A weaker muscle can create more work than a stronger muscle, if the moment arm is longer.

In a study by Otis et al., it was shown that the FB had a greater effect on external rotation of the navicular bone relative to the talus as well as on valgus at the subtalar joint compared with the FL [28]. If the FB has greater effect on externa rotation of the navicular relative to the talus, this indicates that the FB is also the stronger evertor, since the tarsal bones are a part of a closed kinematic chain where motion of one segment affects all the others [29]. The effect FB has on abduction of the foot might be of great importance to the hindfoot stability. This aligns well with the clinical observation of the varus hindfoot in the case of FB injury.

### Clinical implications

Tendon transfers of both the FL and FB are quite commonly performed, since the fibularis tendons risk rupture during a supination injury as well as in the case of ligamentous laxity [2, 3]. It is not uncommon for the surgeon to find severe longitudinal tears, especially in the FB, leading to the excision of one of the fibularis tendons and maybe an additional tendon transfer. Tendon transfers of the fibularis muscles have also been used in neurological conditions to, for example, correct pes equinus. In addition, the FB has been suggested as a graft for treatment of ankle instability, while the FL has been used to replace the cruciate ligaments of the knee. The surgeon must have knowledge of the muscles’ effect of the hindfoot stability when choosing the most suitable tendon graft. Following the more recent evidence of the FBs important role in the stability of the foot, we recommend that the surgeon should try to avoid sacrificing this muscle–tendon unit.

## Conclusion

These results challenge the long-held assumption that in comparison to the FB, the FL is the stronger and more critical muscle for hindfoot eversion. Prior studies assume that muscle–tendon interaction and importance are based purely on muscle volume, leading to a conclusion that the FL would have a greater work output. Clinical observations led us to test this hypothesis through the dissection and measurement of 16 lower limbs, providing a strong indication that the muscle architectures of the FL and FB muscles are similar. Thus, muscle architecture is unlikely to be the reason for their difference in performance. Both muscles are bipennate with similar insertional angles and a proximal part of the muscle consisting of parallel fibres. It is clear that despite the similarities in muscle architecture, the biomechanical properties of the two muscles are different. Biomechanical studies examining moment arms indicate that the FB has a larger effect on external rotation of the foot. Future studies will be needed to describe the difference in function between the two muscle–tendon units in more detail.

## Data Availability

The data that support the findings of this study are available on request from the corresponding author, AES.

## Abbreviations

FL: Fibularis longus
FB: Fibularis brevis
PCSA: Physiological cross-sectional area
